# Differences between proximal bone remodeling in femoral revisions for aseptic loosening and periprosthetic fractures using the Wagner SL stem

**DOI:** 10.1186/s12891-021-04062-6

**Published:** 2021-02-17

**Authors:** Gábor Friebert, Csaba Gombár, András Bozó, Ilona Polyák, Ádám Brzózka, Krisztián Sisák

**Affiliations:** 1 Department of Orthopaedics, University of Szeged, Szeged, Hungary; 2 Department of Radiology, University of Szeged, Szeged, Hungary

**Keywords:** Hip prosthesis, Revision arthroplasty, Bone-prosthesis Interface, Bone remodeling, Periprosthetic fractures, Prosthesis loosening

## Abstract

**Background:**

Monoblock taper fluted stems have been reliably used to treat proximal femoral periprosthetic fractures (PFF) and femoral aseptic loosening (AL). Although proximal femoral remodeling has been observed around the Wagner Self-Locking (SL) stem, the exact characteristics of this process are yet to be established. Our aim was to compare the remodeling that takes place after femoral revisions for PFF and AL.

**Methods:**

Consecutive patients between January 2015 and December 2017 undergoing femoral revision using the Wagner SL stem for PFF or AL without an extended trochanteric osteotomy (ETO) or bone grafting were selected from our database. Radiological follow-up was performed using plain antero-posterior hip radiographs taken postoperatively and at 3, 6, 12 months and at 24 months. The Global Radiological Score (GRxS) was utilized by four blinded observers. Intra and interobserver variability was calculated. Secondary outcome measures included the Oxford Hip Score and the Visual Analog Scale for pain.

**Results:**

We identified 20 patients from our database, 10 PFF and 10 AL cases. The severity of AL was Paprosky 2 in 2 cases, Paprosky 3A in 2 cases and Paprosky 3B in 6. PFF were classified as Vancouver B2 in 7 cases and Vancouver B3 in 3 cases. Patients undergoing femoral revision for PFF regained 89% (GRxS: 17.7/20) of their bone stock by 6 months, whilst patients with AL, required almost 2 years to achieve similar reconstitution of proximal femoral bony architecture 86% (GRxS: 17.1/20). Inter-observer reproducibility for numerical GRxS values showed a “good” correlation with 0.68, whilst the intra-observer agreement was “very good” with 0.89. Except immediate after the revision, we found a significant difference between the GRxS results of the two groups at each timepoint with pair-wise comparisons. Functional results were similar in the two groups. We were not able to show a correlation between GRxS and functional results.

**Conclusions:**

Proximal femoral bone stock reconstitutes much quicker around PFF, than in the cases of AL, where revision is performed without an ETO. The accuracy of GRxS measurements on plain radiographs showed good reproducibility, making it suitable for everyday use in a revision arthroplasty practice.

**Supplementary Information:**

The online version contains supplementary material available at 10.1186/s12891-021-04062-6.

## Background

Severe bone loss in the proximal femoral metaphysis remains one of the biggest challenges in reconstructive hip surgery. The majority of patients develop proximal bone defects due to aseptic loosening (AL). The most common periprosthetic femur fractures (PFF) (Vancouver B2 and B3) present a similar reconstructive dilemma, where the proximal femur can no longer be used for anchoring the new implant. Removal of the previous implant can also contribute to further bone loss during revision surgery. Autologous bone grafting has limitations in terms of bone available. Using allografts (both morselized and structural) is not without risk, and long-term outcome is unknown with regards to structural grafts.

Taper fluted nonmodular diaphyseally fixed uncemented stems have been proven to be clinically effective in these patient groups [1, 2]. According to the advocates of this stem design, after initial mechanical fixation during surgery, relatively quick biological fixation is achieved by the mechanical stability, the low modulus of elasticity and the grid blasted titanium surface, which promotes bony ongrowth. There is no rigid modular coupling, which might slow down proximal bone restoration. Despite bypassing compromised bone stock proximally, there is no stress shielding in this region, on the contrary, there is predictably proximal new bone formation [3, 4]. This phenomenon does occur with or without a proximal extended trochanteric osteotomy (ETO) or fracture. The exact timeframe and characteristics of this process is unknown.

Assessing fracture healing on radiographs is a subjective process. Evaluating bone restoration is perhaps even more so. Historically both quantitative [5] and qualitative measurement options [1] exist for describing bone restoration in the femur. None of these are easily applicable for both PFF and AL scenarios. Several attempts have been made to objectively describe bone remodeling, although most of the attempts use arbitrary scales. Isacson et al. [6] used a scale from 0 to 3 (0 = no new bone; 1 = some indication of new formation; 2 = cancellous bone surrounding the stem; and 3 = large areas of cortical bone adjacent to the stem surface). Alternatively the presence of residual osteolytic areas can registered according to the work of Böhm and Bischel [7] as increasing defects, constant defects or osseous restoration. Recently more robust and reproducible scoring systems have been introduced to describe bone remodeling. The Global Radiological Score (GRxS) [8] summarizes two previously validated scores, the secondary bone stock (SBS) [9] and osseointegration–secondary stability (O-SS) [10] scores.

The aim of this study was to determine and compare the characteristics and timeframe of bone remodeling around the Wagner Self-Locking (Wagner SL, Zimmer, Warsaw, IN) monoblock stem in revisions for femoral AL and PFF. Our working hypothesis was that there is a distinct difference between the speed of bone stock recovery in the two groups, with AL cases showing a slower recovery process. We also aimed to investigate whether there is a correlation between clinical outcomes and bone regeneration, hypothetising that quicker bone remodeling results in better function.

## Methods

Consecutive patients undergoing revision total hip replacement (THR) between January 2015 and December 2017 utilizing the Wagner SL stem at the Department of Orthopaedics, University of Szeged, were chosen from our prospectively collected revision hip database to be included in the study. According to the indication for femoral revision, the patients were subdivided into AL-group and a PFF-group. Revision procedures for AL were classified according to the Paprosky classification [11]. Patients undergoing revision for PFF were classified according to the Vancouver classification system [12]. The femoral bone loss in PFF patients was also classified using the Paprosky classification usually reserved for AL, as the periprosthetic femur fractures were deemed to represent a deficient proximal femur, just like one encounters in AL. Patients undergoing femoral revisions for other indications (instability, infection, etc.) were excluded. Within the AL-group, only patients where an endofemoral approach was utilized were included. Patients, who had an ETO [13, 14] or where a transfemoral approach was used for acetabular access, component removal or varus remodeling were also excluded.

**Fig. 1:**
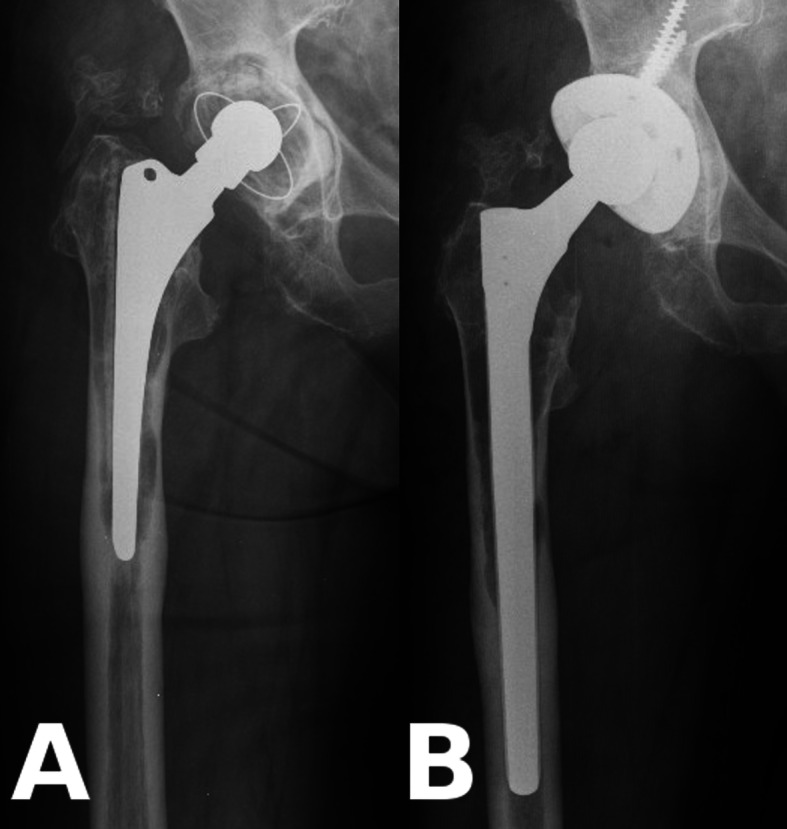
Example for aseptic loosening of the stem. **a**: Preoperatively massive, Paprosky 3B type resorption of proximal femoral bone stock around a cemented stem. **b**: Wagner SL stem fixed in the healthy diaphysis below the lytic region without an ETO (Patient 11)

**Fig. 2:**
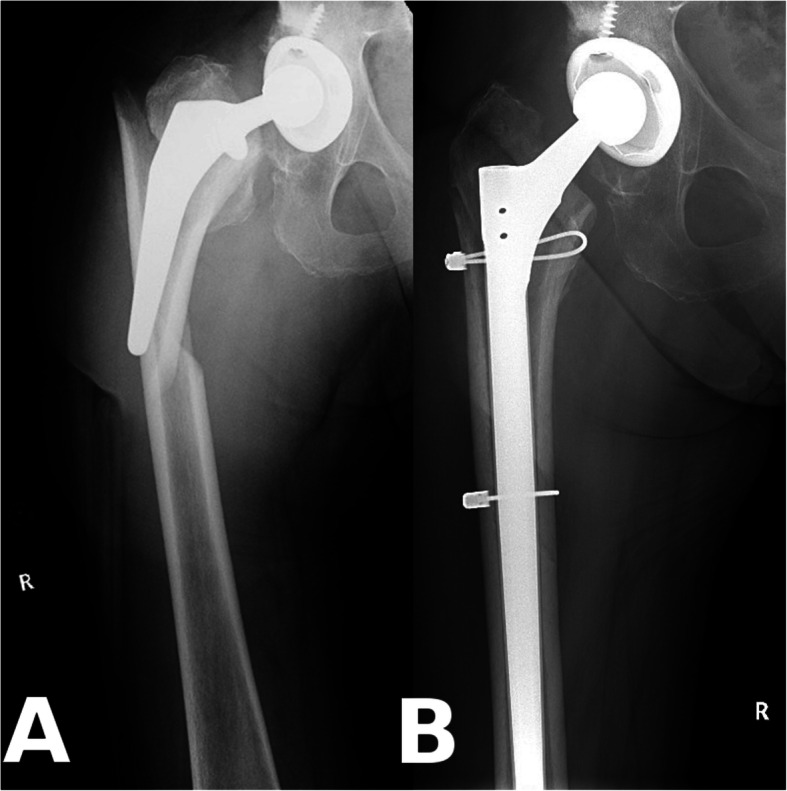
Example for periprosthetic femur fracture. **a**: Vancouver B2 type periprosthetic fracture with component loosening around a cementless stem; **b**: Anatomical reduction with two cables around a Wagner SL stem (Patient 10)

All operations were performed by the senior author, with the patient in the lateral decubitus position, utilizing a posterolateral approach. Procedures were performed under general anaesthesia. The technique was endofemoral in all AL cases (Fig. 1), whilst fractures were treated with either provisional fixation (with clamps and/or wires and an endofemoral technique) or with distal preparation first and proximal reconstruction after revision stem implantation (Fig*.* 2). A prophylactic wire was used in all PFF cases [15]. Cables and/or cerclage wires were used for fixation of fracture fragments. Trochanteric plates were not required in these PFF cases. After the removal of the components and any cement or intramedullary granulomatous tissue, cannulated power reaming was utilized when required, whilst the final femoral preparation was done manually, prior to trialing and the implantation of the Wagner SL stem. An image intensifier was used in all cases. Supplementary bone graft was never used. Routine thromboprophylaxis was administered using Thrombo-Embolus Deterrent Stockings (TEDS) and low-molecular-weight heparin (LMWH) during hospitalization and 30 days thereafter. Antibiotic prophylaxis included intravenous 1.5 g of cefuroxime administered immediately preoperatively and continued for the first 24 h with two additional doses of 750 mg. Passive range of movement exercises were started 24 h after the operation, with touch-toe weight-bearing for 6 weeks. Partial weight-bearing was started 6 weeks postoperatively with 30 kg and increased by 15 kg per week.

Patients were clinically and radiologically followed up for a minimum of 24 months, with follow-ups at 3, 6, 12, 24 months and yearly thereafter.

### Radiological follow-up

Standard antero-posterior (AP) pelvis, AP and lateral radiographs of the operated hip and femur were performed for all patients on the first postoperative day and at 3, 6, 12 months and at 24 months. All postoperative and subsequent follow-up radiographs were performed at the Department of Radiology, University of Szeged following an identical protocol for all patients. Patients were positioned supine, with their feet together. The roentgen tube was positioned at the level of the symphysis, 1 m above and perpendicular to the table. Measurements were performed twice by four doctors: a Consultant Radiologist, a Radiology Regsitrar, a Consultant Orthopaedic Surgeon and by the first author (Orthopaedic Registrar), neither of whom were involved in the operations. All four observers were blinded to the identity of the patients and the date of follow-up radiographs. Intra and interobserver variability was also calculated.

### Measuring bone restoration

Primary outcome measure was the change of the proximal femoral bone stock assessed by using the GRxS [8] with a view to compare the two different indications (AL vs. PFF). The GRxS is the sum of the SBS [9] and the O-SS [10] scores.

**Fig. 3:**
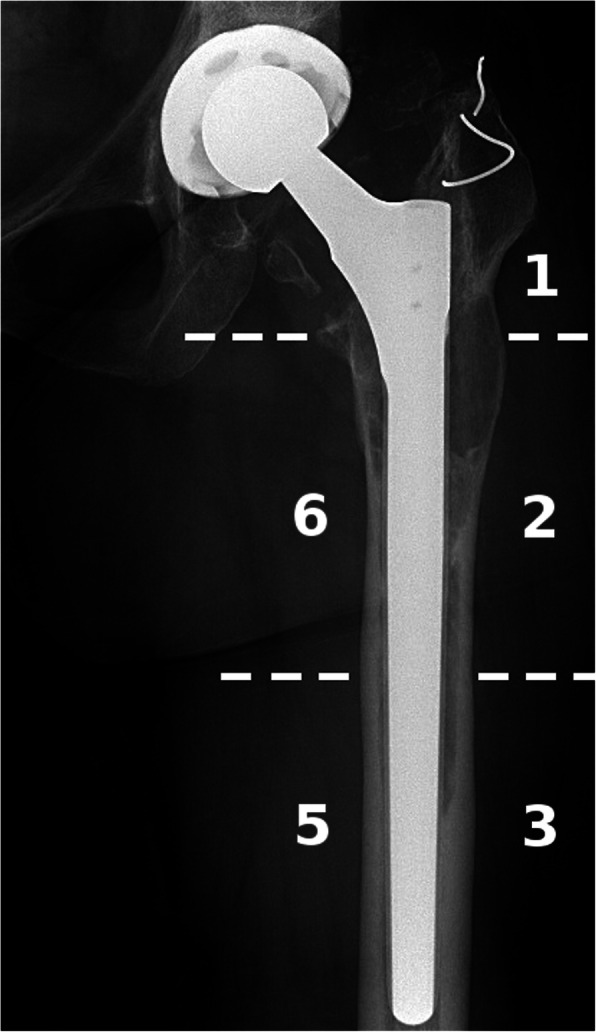
Example for evaluation of Gruen zones on AP radiograph (Patient 18)

Both the SBS score and the O-SS score utilize AP radiographs of the affected hip and use the well-established Gruen zones [16], namely zone 1,2,3,5 and 6 (Fig*.* 3).

The SBS score gives each zone a numerical designation, whilst considering cortical thickness, bone density and cortical bone defects. The SBS scoring system is shown in Table 1.

**Table 1:** Evaluation of SBS scoring of the bone stock by Gruen zones

Score	Bone stock evaluation
**+ 4**	no damage or complete regeneration (density and thickness)
**+ 2**	moderate damage: decreased thickness or density or defects < 10 mms
**0**	severe damage: decreased thickness and density or defects > 10 mms
**-2**	major damage in density and thickness or cortical lysis

The sum of the measurements of the 5 zones are used to create a cumulative value from − 10 to 20. The secondary bone stock is designated very good (20–18), good (16–14), average (12–10) or poor (< 10).

The O-SS score examines the proximal (zone 1,2 and 6) and distal femur (zone 3 and 5) separately. The O-SS evaluation system of radiolucent lines is presented in Table 2.

**Table 2:** Evaluation of O-SS scoring around the stem by femoral parts

Score	Bony bed evaluation
**10**	no radiolucent line
**7**	radiolucent line < 50%
**4**	radiolucent line > 50%

The osteointegration and secondary stability is designated very good (20–18), good (14), average (11) or poor (5–8) (weighting is used by adding 1 point for good proximal bone but some distal radiolucent lines (+ 1) and deducting 3 points for significant proximal radiolucent line scores regardless of distal integration (− 3). To calculate GRxS, one simply gives each very good a 10, good an 8, average a 5 and poor a 2, for both the SBS and O-SS and then adds them together. GRxS is very good (20), good (18–16-15), average (13–12) or poor (≤10) [8].

**Fig. 4:**
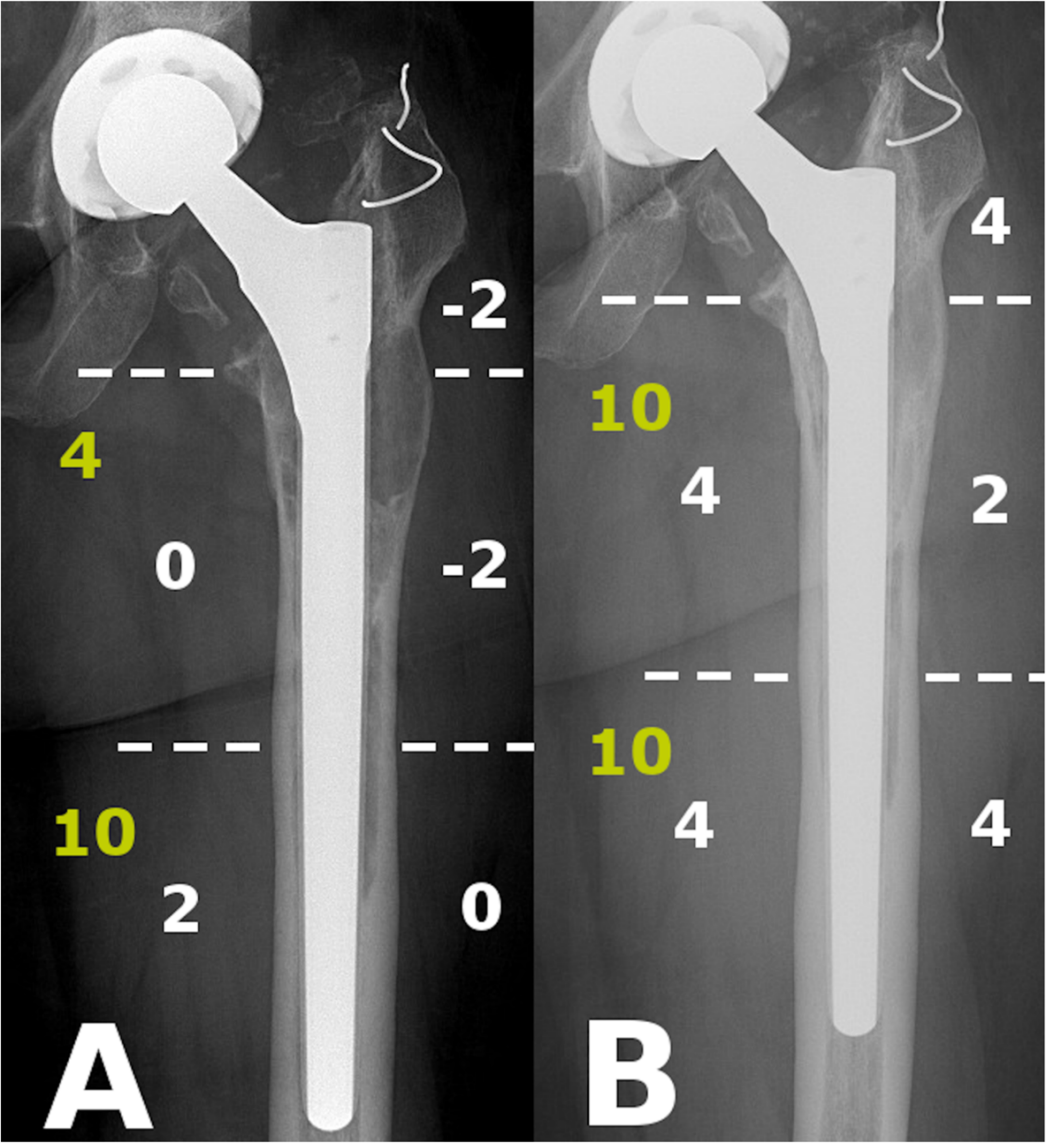
Example for SBS (white) and O-SS (yellow) measurements in AL group. **a**: IBS -2, O-SS 14 immediate after the revision, GRxS: 4; **b**: SBS 18, O-SS 20 at latest follow-up, GRxS: 18 (Patient 18)

**Fig. 5:**
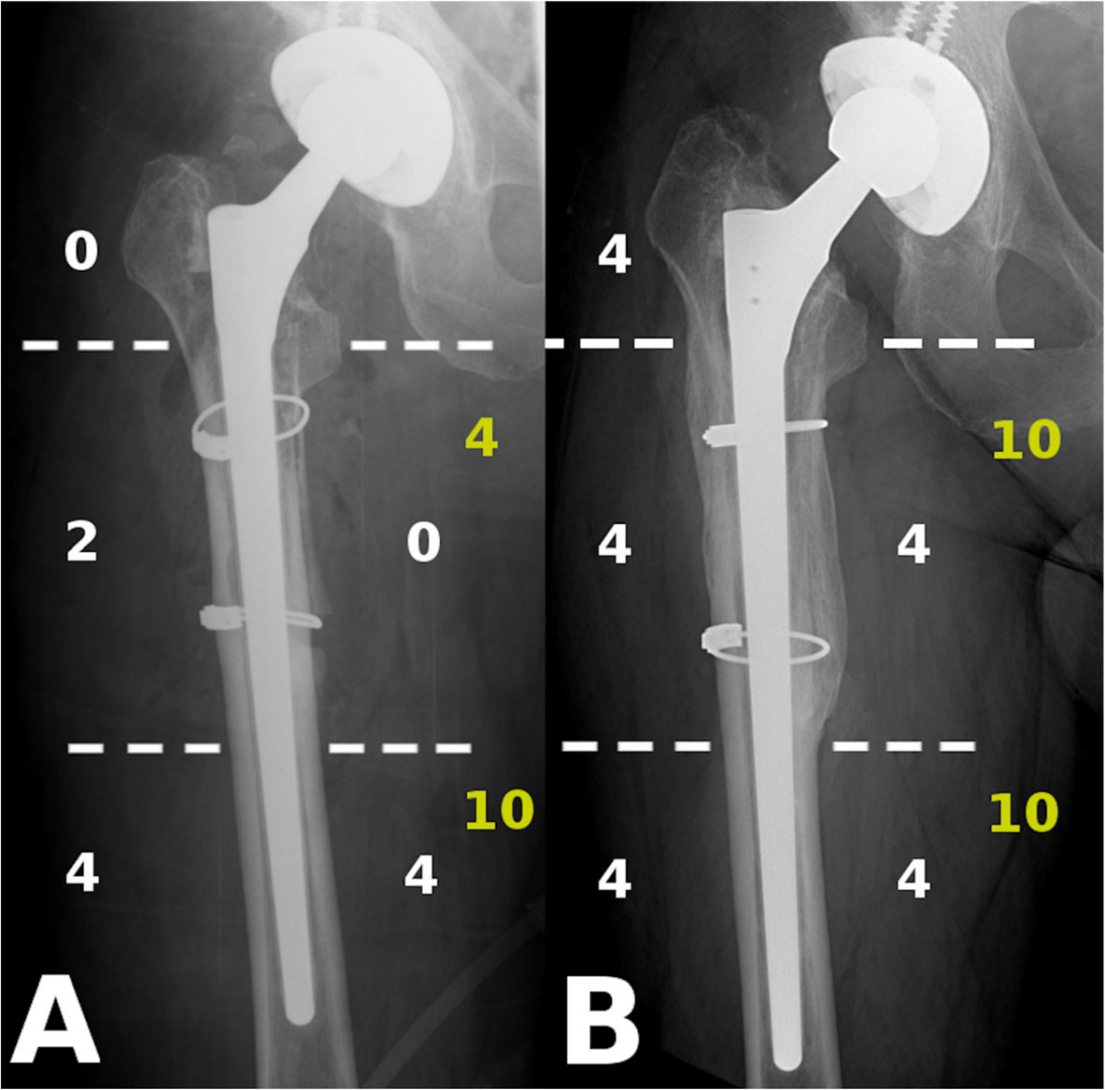
Example for SBS (white) and O-SS (yellow) measurements in PFF group. **a**: IBS 10, O-SS 14 at first immediate after the operation, GRxS: 13; **b**: SBS 20, O-SS 20 at latest follow-up, GRxS: 20 (Patient 07)

The evaluation process is illustrated with radiographs Figs. 4 and 5.

**Fig. 6:**
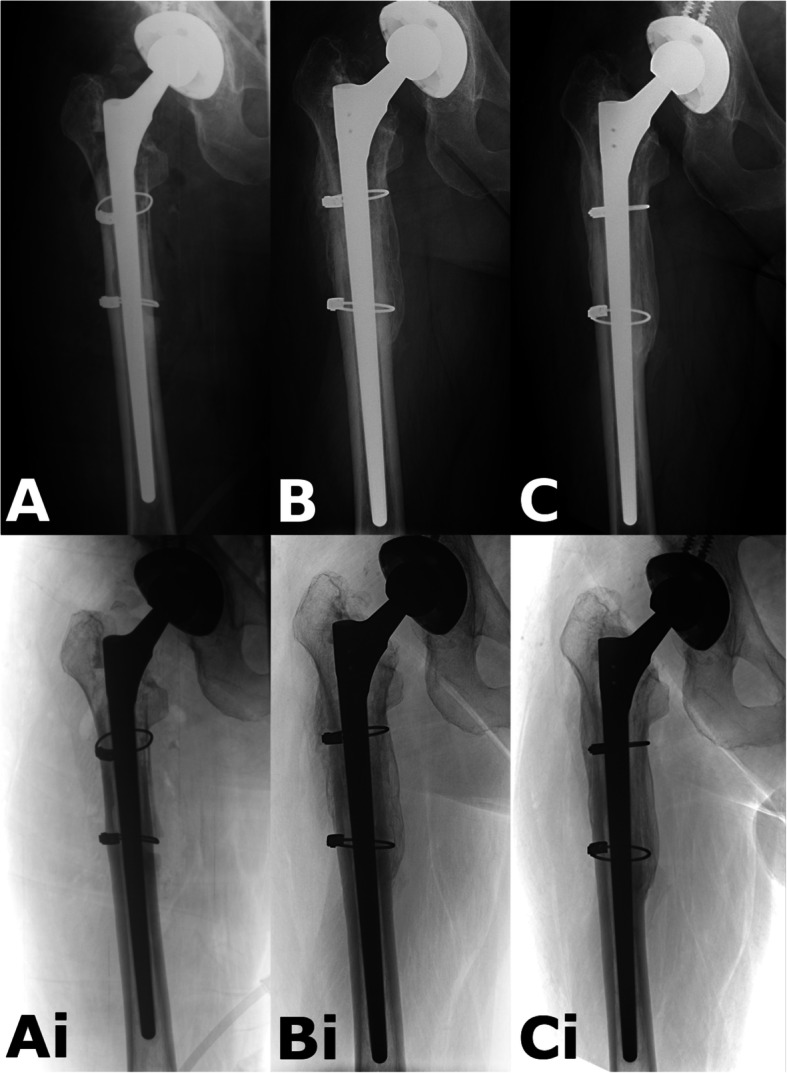
Example of a PFF case. **a**: Immediate postop radiograph after revision. The poor quality of the proximal bone stock is unequivocal. **b**: obvious new bone formation seen at 6 month follow-up. There is some subsidence. **c**: Radiograph 2 years after the operation. Complete reconstitution of bone stock. Ai-Bi-Ci: Shows the same pictures in inverted view (Patient 07)

**Fig. 7:**
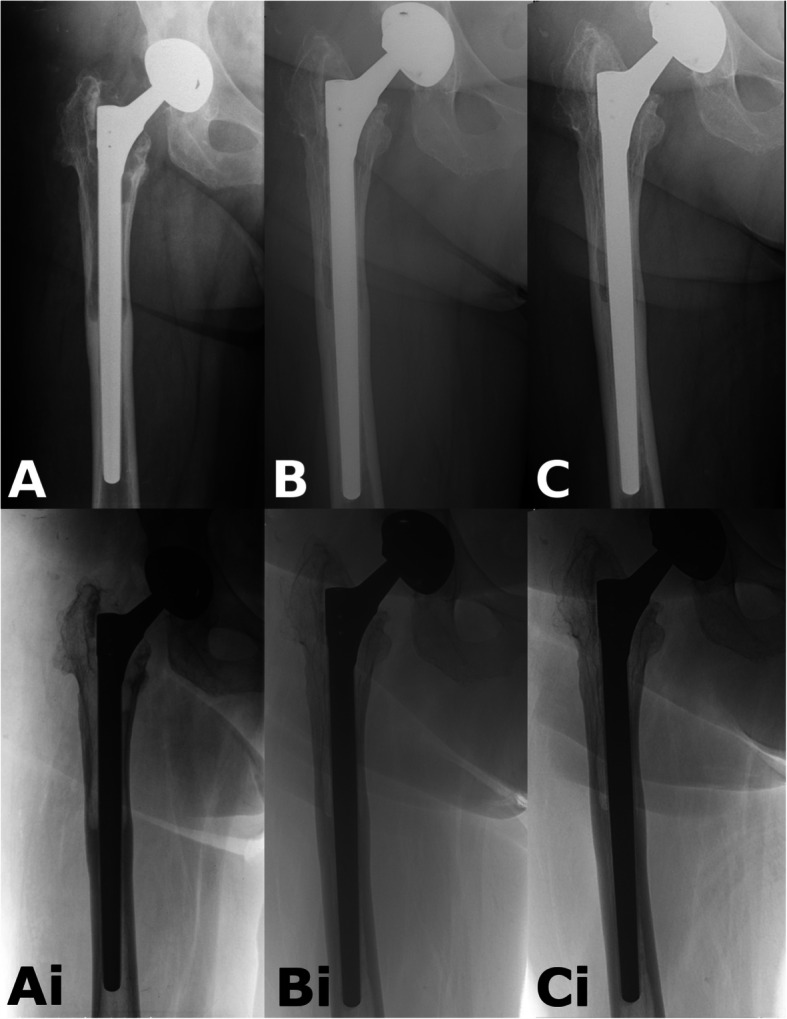
*Example for AL case.* **a**: Postoperative radiograph after revision. Obvious proximal femoral lysis. **b**: After 12 months there is mild subsidence, and continuous bony remodeling. **c**: Almost complete reconstitution of bone stock at 2 years. Ai-Bi-Ci: Shows the same pictures in inverted view (Patient 17)

The immediate postoperative radiographs were compared with those performed at 3, 6, 12 and 24 months to examine bone restoration. Inverted radiographs can aid assessing bone defects and bone quality (Figs. 6 and 7).

The Wagner SL stem is a straight stem without a bevel, thus longer stems can abut to the anterior cortex, due to the normal anterior femoral bow. Bone loss due to the eccentric position of the tip of the stem in the femoral canal has been described and can be evaluated on the lateral view according to Zalzal et al. [17]. The central position of the stem was checked with radiograph intraoperatively as well from lateral view. We utilized only the AP radiographs for measuring bony remodeling, the tip of the stem was not separately assessed on lateral views.

Radiological measurements were performed using the GEPACS software (General Electric Company Healthcare, Chicago, Illinois, USA).

### Secondary outcome measures

Basic demographic data (age, gender, body mass index - BMI) and time to revision were collected. Perioperative parameters recorded included: duration of surgery, type of anesthesia, intraoperative blood loss, transfusion requirement and length of stay.

Clinical examination included grading the pain using the Visual Analogue Scale (VAS), and using the Oxford Hip Score (OHS) as our preferred patient reported outcome measure.

### Statistical analysis

We performed the statistical comparison of demographic data between the AL and PFF groups with Student’s two sample t-test for continuous variables (age, time to revision, length of surgery, blood loss, BMI, length of stay) and Fisher’s exact test for discrete variables (gender, cup revision rate).

We used the Wilcoxon test to compare OHS and VAS results (non-parametric data), two sample or signed design as required.

We analysed the intra-, and interobserver agreement between the numerical GRxS results with intraclass correlation coefficient (ICC) test. For the categorical GRxS results we performed Cohen’s-Kappa for intraobserver and Fleiss-Kappa for interobserver reliability.

For the statistical analysis of the GRxS results, we used both the numerical results and the categorical evaluation “very good”, “good”, “average” and “poor”. The patients were grouped accordingly.

We compared the GRxS measurements between the different timepoints with the Friedman test, KendallW was calculated and for the post-hoc analysis we used the paired Wilcoxon signed-rank test.

For assessing the correlation between GRxS with OHS and VAS values we made Spearman’s rank correlation test.

The significance level was determined at 5% (α = 0,05).

Statistical analyses were performed using the R software (version 3.6.2; The R Foundation for Statistical Computing, Wien, Austria).

Our observational study has obtained the approval of the Clinical Research Coordination Office of the University of Szeged, with the registration number: 3/2019-SZTE. Written consent was attained from all patients involved.

## Results

From our prospective hip revision database, 39 patients were identified who underwent stem revision using the Wagner SL stem, during the above mentioned period. Twenty patients matched our inclusion criteria for diagnosis and surgical technique, and had a minimum of 24 month follow up.

They were divided into two groups according to the reason for revision: 10 patients had a PFF and 10 patients had stem revision for AL. The severity of aseptic loosening was classified in the AL group as Paprosky 2 in 2 cases, Paprosky 3A in another 2 cases and Paprosky 3B in the other 6. In the PFF group the fracture was classified as a Vancouver B2 in 7 cases and Vancouver B3 in 3 cases. The Paprosky classification of PFF cases showed 7 cases of Paprosky 3A and 3 cases of 3B femoral defects. Six out of the seven VB2 cases were classified as P3A, whilst only one of the three VB3 cases was P3A, the rest were P3B. The comparative classification of PFF patients can be *found in* Tables 3 and 4.

**Table 3:** Paprosky classification of AL and PFF groups

	AL	PFF
**P2**	2	0
**P3A**	2	7
**P3B**	6	3

**Table 4:** Comparison of the Paprosky and Vancouver classifications of PFF group

	VB2	VB3
**P3A**	6	1
**P3B**	1	2

The revision was a first revision in 15 cases, second revision in 3 cases and a third revision in 2 cases. The side distribution was even with 50% (10/20) left and 50% (10/20) right. 65% (13/20) of the patients required a blood transfusion, nine of them intraoperatively.

Detailed patient demographics and peri-operative data can be found in Table 5.

**Table 5:** Peri-operative data and demographics of AL cases and PFF patients

	Sum. Mean	AL	PFF	***p***-value	95% CI
**Age** (years)	66 (41–78)	65 (41–78)	66 (51–78)	0.8736	9.815032; 8.415032
**Gender** (Female)	55% (11/20)	35% (7/20)	20% (4/20)	0.3698	0.03005364; 2.46429183
**BMI** (kg/m^2^)	31.3 (17.8–44.3)	34.1 (27.2–44.3)	28.5 (17.8–40.6)	0.06305	0.3417606; 11.6617606
**Time to revision** (months)	144 (3–316)	173 (75–316)	115 (3–264)	0.098	−11.76233; 127.16233
**Surgery length** (minutes)	175 (100–260)	163 (100–245)	187 (120–260)	0.1917	59.89746; 12.89746
**Cup revision** (Y/N)	13 / 7	9 / 1	4 / 6	0.05728	0.9487882; 684.4235629
**Bloodloss** (mls)	800 (0–1800)	600 (0–1500)	1000 (300–1800)	0.06291	− 822.03633; 24.03633
**Length of stay** (days)	12 (6–23)	9 (6–13)	15 (10–23)	0.00053	−8.997446; −3.002554

*Sum. Mean* Summarized means; *95% CI* Confidence Interval 95%; ranges in the parentheses

### OHS/vas

Clinical follow-up included, in terms of patient reported outcome measure, the OHS, and for pain specifically the VAS. Preoperative values were compared with the ones at the latest follow-up.

In the AL group OHS values improved significantly from an average preoperative value of 13 points (3–25), to a latest follow-up value of 30 (15–41) (Wilcoxon rank sum test with continuity correction; *p* = 0.005857). As periprosthetic fracture are mostly acute events, measuring the OHS preoperatively is inappropriate, due to pain and restricted mobility (or applied skeletal traction). Comparing postoperative values, PFF patients scored higher 35 (14–48), although the difference was not significant (Wilcoxon rank sum test with continuity correction; *p* = 0.2892).

VAS values were analyzed in a similar way. In the AL group preoperative values averaged 7.3 (4–10), whilst at the latest follow-up the average was 2.6 (0–7) (Wilcoxon signed rank test with continuity correction; *p* = 0.005603). PFF patients scored 1.9 (0–7) at the latest follow-up. AL vs PFF comparison did not show a significant difference (Wilcoxon rank sum test with continuity correction; *p* = 0.7017).

### GRxS

In terms of inter-observer reproducibility, for the four independent examiners who performed the measurements, the results showed “good” reproducibility, with ICC 0.68 (*p* < 0.001; 95% CI 0.57–0.77). For categorical variables the Fleiss-Kappa showed a “good” correlation with 0.548 (p < 0.001).

The intra-observer agreement for numerical GRxS values was considered “very good” ICC 0.89 (p < 0.001; 95% CI 0.84–0.93), whilst for categorical variables the weighted Cohen-Kappa was also “very good” with Kappa 0.84 (p < 0.001).

**Fig. 8:**
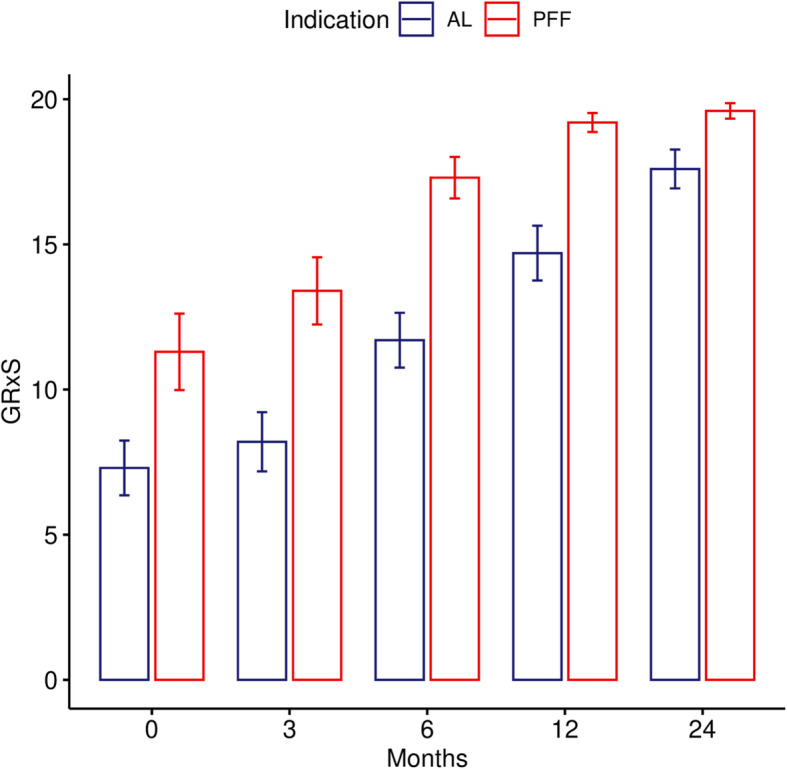
The GRxS means of AL and PFF groups at the follow-up timepoints. There is a significant difference between the results of the groups at each timepoint (except immediate after the operation) with paired Wilcoxon signed-rank test (0 = immediate after the operation (*p*-value = 0.08198), after 3 months (*p*-value = 0.03412), 6 months (*p*-value = 0.008492), 12 months (*p*-value = 0.0213), and 24 months (*p*-value = 0.01788))

At the immediate postoperative follow-up in the AL group 9 patients had a poor (6.7 points), one an average (13 points) GRxS evaluation, whilst for patients in the PFF group 6 had a poor value (8.5 points) and 2 each had an average (13 points), and good (18 points) categorization. None of the patients had a very good designation at this point. At the latest follow-up (minimum of 2 years) the GRxS scores for the AL group showed average category in one (13 points), good in 7 (17.6 points) and very good in 2 cases (numerical 20). For the PFF patients 2 patients had a good rating (numerical 18), whilst the remaining 8 had a very good rating (20 points). The differences between the groups in different timepoints are illustrated in Fig. 8.

**Fig. 9:**
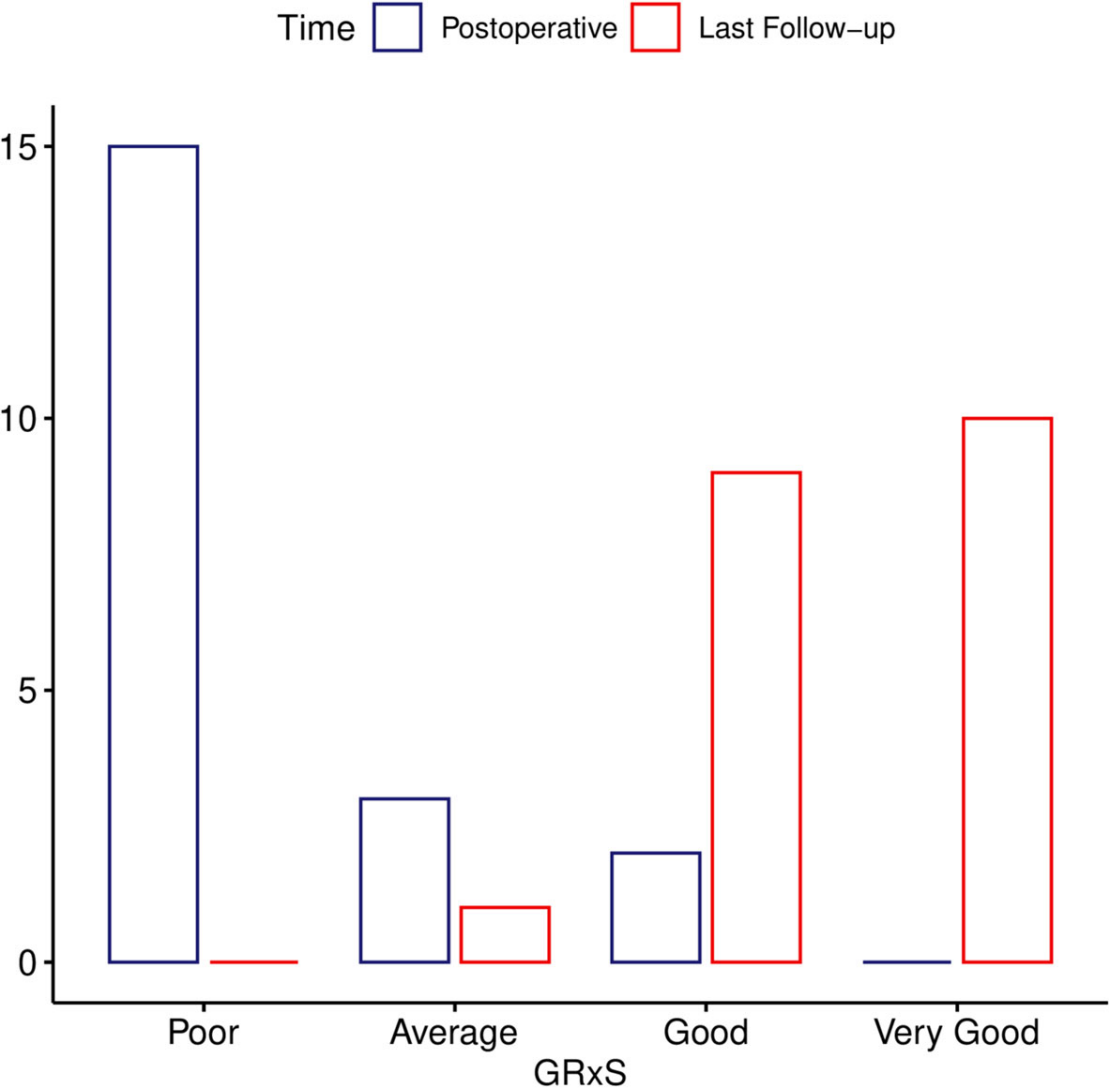
Change of GRxS group classification between immediate postoperative and last follow-up measurements

The change of GRxS group classifications between first and last measurements is illustrated in Fig. 9*.* The bony changes are demonstrated with radiographs between the follow-up sessions on Figs. 6 and 7.

We analyzed the GRxS measurements between the different timepoints statistically. We found a significant difference between the results at each 5 timepoints (Friedman x^2^ = 70.812; *p* < 0.001; KendallW = 0.88515/large/). For the pair-wise comparisons we used paired Wilcoxon signed-rank test. Except immediate after the operation, we found a significant difference between the two groups at each timepoint.

The comparison between the groups is illustrated in Fig. 8.

As illustrated, patients undergoing femoral revision for PFF using the Wagner SL stem, can expect to regain 89% (17.7/20) of their bone stock by 6 months, whilst patient having a revision for AL, require almost 2 years to achieve nearly similar reconstitution of proximal femoral bony architecture 86% (17.1/20).

Finally we compared the correlation of GRxS with OHS and VAS values. We did not find a significant relationship between these parameters with Spearman’s rank correlation test (rho = − 0.2 and − 0.1; *p* > 0.05).

### Complications

There was one early dislocation, which was successfully treated with a closed reduction. There was one intraoperative greater trochanter fracture, which went on to unite in 6 months.

None of the femoral components required a revision within the follow-up period. The overall survivorship therefore was 100% for the stems, with femoral revision being the endpoint.

## Discussion

The treatment of femoral AL and PFF has undergone a paradigm change over the last 30 years. Although impaction bone grafting and the implantation of a long cemented stem remains a viable option, with a good track record in some centers [18], the mainstay of treatment has been the use of uncemented revision stems. Cylindrical, nonmodular cobalt-chromium uncemented stems [19] have given way to tapered fluted titanium stems both in a nonmodular [20–22] and modular configuration [23]. Both provide reliable long-term functional results, with nonmodular implants having the disadvantage of potential early subsidence and the lack of proximal modularity (thus instability), but the advantage of being more elastic, osteointegrating quicker whilst avoiding the risk of coupling failure.

Osseointegration of uncemented implants is required for long-term stability and appropriate joint function. In our study we have demonstrated that proximal femoral bone restoration takes place reliably, both after revision for PFF and for AL, using a monoblock taper fluted revision stem. However, there is a distinct difference in the timeframe of this process in the two patient groups, with PFF patients taking only 6 months to regain about 90% of the bone stock, whilst AL patients require more than 2 years to achieve nearly the same.

Our findings are comparable with other studies. Some papers reported similar timeframe (4–6 months) of fracture healing in PFF cases and also in cases where an ETO was performed [1, 22, 24].

Sandiford et al. [2] reported in patients with P2 and P3 type defects encouraging proximal femur bony regeneration after 2 years.

Measuring radiological bone quantity and assessing bone quality on plain radiographs is a subjective process. Determining the radiological features of cementless arthroplasty components has been a topic of ongoing research for decades.

Canovas et al’s [8] designed a complex and detailed scoring system. The radiological evaluation of remodeling has thus became more accurate.

Canovas et al. [9] in their first study of the topic created a new scoring system from a different perspective that used the former (Engh et al. [25]) radiological signs to evaluate the bony remodeling after stem revision. The initial bone stock (IBS) and SBS scores can determine the bone stock around modular revision stems [9].

Roche et al. [10] in their study have found that the measurement of radiolucent lines using the O-SS score can be a reliable method evaluating the osseointegration and the secunder stability of extensively porous coated (scratch fit cylindrical) uncemented stems. They have found a significant difference between their method and Engh’s method, and have found no correlation between stem stability and secondary subsidence.

Canovas et al. [8] finally merged these two scoring systems to form the GRxS, which was the scoring system that we utilized in our study. In Canovas’s medium term study, they evaluated a modular taper fluted porous coated stem (Revitan, Zimmer, Warsaw, IN) which was used for revisions in aseptic loosening cases. There was no bone graft used during the procedures, but in two thirds of the cases an ETO was performed. They found a significant relationship between the GRxS score and the functional outcomes.

In our study we did not find a statistical relationship between GRxS and OHS or VAS parameters.

Gutierrez et al. [1] examined the bone regeneration after stem revision using the Wagner SL stem. They reported 92.3% stem survival with the most common failure mechanism being subsidence and instability. They observed more pronounced bone remodeling and cortical thickening when there were no major proximal femoral defects. The bone formation was most pronounced at the site of PFF fractures or ETO, which in a sense is similar to our finding that bone remodeling is significantly faster if there is a fracture present.

In AL proximal femoral stress shielding and bone atrophy following total hip replacement, with time will leave very little viable cancellous bone proximally, with thinned and often eroded cortices. Callus formation after fractures on the contrary seems to accelerate bone remodeling. Whilst endofemoral bony apposition and remodeling takes place around a taper fluted stem regardless of preoperative diagnosis, pronounced periosteal bone formation is seen in fracture cases, especially if the required reduction and retention (osteosynthesis) technique respects the blood supply of bone fragments. Proximal femoral callus increases the contact area between implant and host bone, thus facilitating load transfer through a larger surface. Fracture pattern and bone quality will influence the required fixation method [26]. Close to anatomical reduction of fracture fragments accelerates the bone remodeling process.

To evaluate the bone remodeling we used a four blinded observer model with two independent measurements each. The GRxS values showed “good” inter-observer reproducibility and “very good” intra-observer agreement for categorical and numerical variables. We analyzed the GRxS measurements between the different timepoints and we found a greatly significant difference between the results at each 5 timepoints. Pair-wise comparisons showed a significant difference between AL and PFF groups at each timepoint, except at the first follow-up. To our knowledge, this paper is the first to compare PFF and AL bone remodeling in such detail using the Wagner SL stem.

The known limitations of our study are the relatively small patient numbers, and the length of follow-up, although other papers examining the same stem have similar numbers, e.g. Zang et al. reviewed 40 hips operated during a much longer, 12-year period [27]. The small patient numbers effected our statistical analysis. The established strengths include, a universal treatment protocol (surgical approach, one surgeon series, postoperative rehabilitation protocol) and the rigorous radiological assessment of the proximal femoral bone stock performed by four independent, blinded observers. The detailed chronological comparison of periprosthetic femoral fractures and aseptic loosening cases is also unique to our study.

Measuring bone stock on plain radiographs is feasible and reproducible. CT scans might provide more detailed information about three dimensional bony remodeling and implant-host bone contact [28]. Further long term assessment is required for detailing the bone remodeling according to preoperative defect category (Paprosky or Vancouver) to help better understand risk factors for delayed osteointegration. Comparison of monoblock and modular stems in regards of bone remodeling would also clarify the indications for the different stem types.

## Conclusions

From our hip revision database we examined femoral revisions using the Wagner SL stem without an ETO or bone grafting. Our main finding is that proximal femoral bone stock reconstitutes much quicker around periprosthetic fractures, than in the cases of aseptic loosening, where revision is performed without an ETO. The accuracy of our measurements on plain radiographs is accurate enough for everyday orthopaedic arthroplasty practice with the use of a GRxS scoring system.

## Supplementary Information

**Additional file 1.**

## Data Availability

The datasets used and/or analysed during the current study are available from the corresponding author on reasonable request.

## Abbreviations

AP: Antero-posterior
AL: Aseptic loosening group
Av: Average
BMI: Body Mass Index
95% CI: Confidence Interval 95%
ETO: Extended trochanteric osteotomy
GRxS: Global Radiological Score
G: Good
IBS: Initial bone stock
ICC: Intraclass correlation coefficient
LOS: Length of stay
LMWH: Low-molecular-weight heparin
mms: Millimeters
O-SS: Osseointegration–secondary stability
OHS: Oxford Hip Score
PFF: Periprosthetic femoral fracture group
Pr: Poor
SBS: Secondary bone stock
SL: Self-Locking
Sum. Mean: Summarized mean
TEDS: Thrombo-Embolus Deterrent Stockings
THR: Total hip replacement
vs: Versus
VG: Very Good
VAS: Visual analogue scale
