# Supplementary material for: Differences between proximal bone remodeling in femoral revisions for aseptic loosening and periprosthetic fractures using the Wagner SL stem
Source: BMC Musculoskelet Disord. 2021 Feb 17;22:201. doi: 10.1186/s12891-021-04062-6 (PMC7890820; doi:10.1186/s12891-021-04062-6)
Supplement: Supplementary file 1 — Additional file 1. [file 12891_2021_4062_MOESM1_ESM.docx]

Additional data files

Figure 1

Figure 2

Figure 3

Figure 4

Figure 5

Figure 6

Figure 7

Figure 8

Figure 9
